# Structural and functional implications of *SLC13A3* and *SLC9A6* mutations: an in silico approach to understanding intellectual disability

**DOI:** 10.1186/s12883-023-03397-y

**Published:** 2023-10-04

**Authors:** Syeda Iqra Hussain, Nazif Muhammad, Salah Ud Din Shah, Fardous Fardous, Sher Alam Khan, Niamatullah Khan, Adil U Rehman, Mehwish Siddique, Shoukat Ali Wasan, Rooh Niaz, Hafiz Ullah, Niamat Khan, Noor Muhammad, Muhammad Usman Mirza, Naveed Wasif, Saadullah Khan

**Affiliations:** 1 Department of Biotechnology and Genetic Engineering, Kohat University of Science & Technology (KUST), Kohat, Khyber Pakhtunkhwa Pakistan; 2 Department of Medical Lab Technology, Kohat University of Science & Technology (KUST), Kohat, Khyber Pakhtunkhwa Pakistan; 3 Department of Zoology, Government Post Graduate College for Women, Satellite Town, Gujranwala, Pakistan; 4 Department of Botany, Faculty of Natural Sciences, Shah Abdul Latif University, Khairpur, Sindh Pakistan; 5 Gomal Center of Biochemistry and Biotechnology (GCBB), Gomal University D. I. Khan, D. I. Khan, Pakistan; 6 Department of Chemistry and Biochemistry, University of Windsor, Windsor, ON N9B 1C4 Canada; 7 Institute of Human Genetics, Ulm University and Ulm University Medical Center, 89081 Ulm, Germany; 8 Institute of Human Genetics, University Hospital Schleswig-Holstein, Campus Kiel, Kiel, Germany

**Keywords:** Intellectual disability, Acute reversible leukoencephalopathy, Christianson Syndrome, Exome sequencing, *SLC13A3*, *SLC9A6*, Molecular dynamics simulation

## Abstract

**Background:**

Intellectual disability (ID) is a condition that varies widely in both its clinical presentation and its genetic underpinnings. It significantly impacts patients’ learning capacities and lowers their IQ below 70. The solute carrier (SLC) family is the most abundant class of transmembrane transporters and is responsible for the translocation of various substances across cell membranes, including nutrients, ions, metabolites, and medicines. The SLC13A3 gene encodes a plasma membrane-localized Na+/dicarboxylate cotransporter 3 (NaDC3) primarily expressed in the kidney, astrocytes, and the choroid plexus. In addition to three Na + ions, it brings four to six carbon dicarboxylates into the cytosol. Recently, it was discovered that patients with acute reversible leukoencephalopathy and a-ketoglutarate accumulation (ARLIAK) carry pathogenic mutations in the *SLC13A3* gene, and the X-linked neurodevelopmental condition Christianson Syndrome is caused by mutations in the *SLC9A6* gene, which encodes the recycling endosomal alkali cation/proton exchanger NHE6, also called sodium-hydrogen exchanger-6. As a result, there are severe impairments in the patient’s mental capacity, physical skills, and adaptive behavior.

**Methods and results:**

Two Pakistani families (A and B) with autosomal recessive and X-linked intellectual disorders were clinically evaluated, and two novel disease-causing variants in the *SLC13A3* gene (NM 022829.5) and the *SLC9A6* gene (NM 001042537.2) were identified using whole exome sequencing. Family-A segregated a novel homozygous missense variant (c.1478 C > T; p. Pro493Leu) in the exon-11 of the *SLC13A3* gene. At the same time, family-B segregated a novel missense variant (c.1342G > A; p.Gly448Arg) in the exon-10 of the *SLC9A6* gene. By integrating computational approaches, our findings provided insights into the molecular mechanisms underlying the development of ID in individuals with SLC13A3 and SLC9A6 mutations.

**Conclusion:**

We have utilized in-silico tools in the current study to examine the deleterious effects of the identified variants, which carry the potential to understand the genotype-phenotype relationships in neurodevelopmental disorders.

**Supplementary Information:**

The online version contains supplementary material available at 10.1186/s12883-023-03397-y.

## Introduction

Intellectual disability (ID) refers to a range of neurodevelopmental abnormalities, and about 2% of children or young people have ID, which is described as having significant deficits in intellectual functioning and adaptive behavior and is associated with an IQ below 70 [1]. Chromosomal abnormalities, such as pathogenic deletions, duplications, or single-gene deficiencies with recessive, X-linked, or autosomal-dominant inheritance, can lead to moderate to severe forms of ID [2]. Mendelian types of ID have been linked to more than 500 genes [3].

ARLIAK (acute reversible leukoencephalopathy with elevated urine alpha-ketoglutarate) is an autosomal recessive condition causing acute reversible neurologic degeneration during a febrile illness. On brain imaging, the disease is associated with transitory leukoencephalopathy and consistently elevated excretion of dicarboxylic acids, particularly alpha-ketoglutarate. The Na+/dicarboxylate cotransporter 3 (*NaDC3*) gene encodes the Na+/dicarboxylate cotransporter 3 (*SLC13A3*), found on the plasma membrane and carries necessary metabolic intermediates into cells [4, 5]. Aside from citric acid cycle intermediates such as succinate and ketoglutarate [6], *SLC13A3* transports additional critical metabolic chemicals into the cell, such as glutathione [7], mercapto succinate, and N-acetyl aspartate (NAA) [8]. *SLC13A3* is essential for cell nutrition and detoxification. Pathogenic *SLC13A3* mutations cause acute reversible leukoencephalopathy (a heterogeneous set of disorders characterized by developmental defects or white matter degeneration) and ketoglutarate accumulation (ARLIAK) [9].

Christianson syndrome (CS) is a neurodevelopmental and progressive neurodegenerative disorder characterized by moderate to severe intellectual disability, epilepsy, mutism, truncal ataxia, hyperkinesis, happy demeanor, and postnatal microcephaly. It is often accompanied by one or more secondary symptoms (such as autistic behavior, eye movement dysfunction, hypotonia, gastroesophageal reflux, low height and weight, high pain threshold, motor regression, cerebellar vermis, and brain stem atrophy as well as neuronal cell loss) [10–12]. *SLC9A6* is one of the most frequently mutated genes connected to X-linked intellectual disability (XLID) [13–15]. The prevalence of CS among X-linked developmental brain disorders is estimated to be between 1% and 2% [12, 16, 17]. Males are disproportionately affected by *SLC9A6* mutations, as with most X-linked disorders, while female carriers typically show no symptoms or a milder phenotype [10, 12, 18]. One of the common pathways to which both genes (*SLC9A6* and *SLC13A3*) are related is the “Transport of inorganic cations/anions and amino acids/oligopeptides” [19].

Molecular dynamics (MD) simulations have emerged as a valuable tool for understanding the effects of mutations on protein structure, function, and dynamics [20–24]. By employing MD simulations, it is possible to gain insight into the structural and functional consequences of the identified mutations in *SLC13A3* and *SLC9A6*, thus elucidating the potential molecular mechanisms underlying the development of ID in the affected families. In addition, these simulations can provide detailed information about changes in protein conformation, stability, and interactions with other cellular components, offering valuable clues about how the mutations may impair protein function [25, 26]. In this study, we performed long-run MD simulations to examine the impact of the novel missense variants found in the *SLC13A3* and *SLC9A6* genes. Our analysis aims to shed light on the likely molecular consequences of these mutations and how they could contribute to the pathogenesis of ID, thereby broadening our understanding of the genotype-phenotype relationship in the context of these neurodevelopmental disorders.

## Methods

### Sample collection

**Fig. 1:**
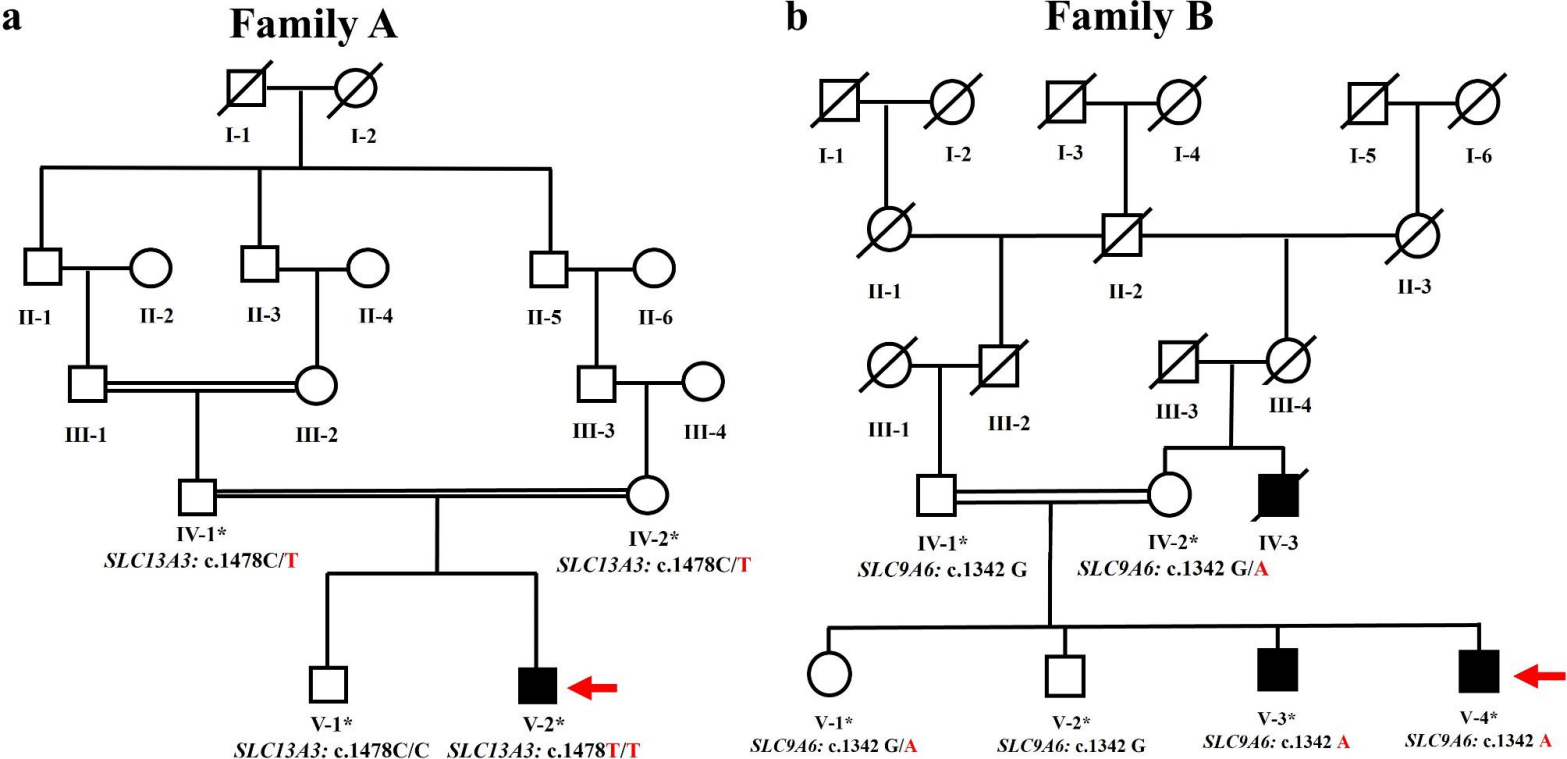
**(a)** Pedigree of Family A showing the autosomal recessive pattern of ID and presenting the unaffected and affected individuals of the family (A) **(b)** Pedigree of Family B showing the X-linked pattern of ID and presenting the unaffected and affected individuals of the family (B) Arrows are representing the DNA samples of the individuals submitted for exome sequencing

Two families (A and B) with autosomal recessive intellectual disability and X- linked intellectual disability were sampled from Billitang, Kohat, and North Waziristan of Khyber Pakhtunkhwa Province, Pakistan. Four available members were recruited in family A, including one affected and three unaffected individuals. Similarly, six available members were investigated, including three affected and three phenotypically unaffected individuals from family B. Information was collected from the adults in both families regarding their family histories, and the pedigrees were constructed using the information provided by the families. No evidence of a family history (both families) of intellectual impairment was found in the pedigree analysis (Fig. 1). After receiving informed written consent, blood samples were collected in BDA vacutainer tubes and stored. DNA was extracted from blood samples using the usual phenol-chloroform procedure and was quantified up to 40ng. The institutional ethical review board of Kohat University of Science and Technology, Kohat, Pakistan, approved the study protocols, and all methods were carried out, strictly following the recommendations of the Declarations of Helsinki.

### Exome sequencing

Genomic DNA was extracted from the patient’s blood samples of both families. First, the exonic regions of all 22,000 human genes were captured by the xGen Exome Research Panel v2 (Integrated DNA Technologies, Coralville, Iowa, USA). After capture, Novaseq 600 was used to sequence all captured regions (Illumina, San Diego, CA, USA). We acquired ≥ 20X coverage in > 98.9% and ≥ 10X coverage in > 99.4% of target sequences. Following the sequencing, the data was analyzed using open-source bioinformatics tools and proprietary software. bcl2fastq v2.20.0.422 (https://emea.support.illumina.com/downloads/bcl2fastq-conversion-software-v2-20.html) was used to convert and demultiplex base call (BCL) sequence files to FASTQ files. Variant calling and annotation followed the alignment of the sequencing data to the GRCh37/hg19 human reference genome was carried out using BWA-mem 0.7.17 (arXiv:1303.3997 [q-bio.GN]) to generate BAM files. BAM files were processed using GATK best practices (GATK v.3.8, broadinstitute.org) for single nucleotide variants (SNV) and small insertions/deletions (indel) variant calling to generate VCF files [27, 28]. For copy number variant (CNV) calling based on depth-of-coverage (DOC) data, Conifer [29] and 3bCNV (https://3billion.io/resources) are used. The Homozygosity (ROH) regions were mapped from the VCF file using AutoMap v1.2 [30].

One of the in-house tools, EVIDENCE, was designed to select variants based on ACMG guidelines and each patient’s phenotype. Variant filtration, categorization, and similarity score for the patient’s phenotype are three significant steps in this approach. For allele frequency estimation, a genome aggregation database (gnomAD, http://gnomad.broadinstitute.org/) and a 3-billion genome database were utilized in the first step. According to ACMG guidelines, gene variations with more than 5% allele frequency were filtered out. Next, the VarSome [31], Human Gene Mutation Database (HGMD) Professional 2022.1, Database of Single Nucleotide Polymorphisms (dbSNP), and ClinVar (https://www.ncbi.nlm.nih.gov/clinvar/) were utilized for the evaluation of variants. Then, each variant concerning disease phenotype was assessed using the ACMG guidelines [32]. Finally, in the third step, the patient’s clinical phenotypes were converted to standardized human phenotype ontology terms (https://hpo.jax.org/) and retrieved to determine the degree of similarity [33, 34] with each of 7,000 rare genetic diseases (https://omim.org/ and https://www.orpha.net/consor/cgi-bin). According to the ACMG guideline, the similarity score between each patient’s phenotype and symptoms related to that disease caused by priority variations varied from 0 to 10. Medical geneticists and physicians then manually evaluate probable alterations and related disorders. Bidirectional Sanger sequencing is used to confirm single nucleotide variants and all indels.

### Segregation analysis

The filtered variants were then subjected to Sanger sequencing to validate the segregation of the genetic variants in the families. The online Primer3 software designed the primers (flanking the variant regions). Two sets of primers were designed:

*SLC13A3*_Forward 5ˊCACACATGCATGGGACTC 3ˊ,

*SLC13A3*_Reverse 5ˊCACTGTGCAGAGAGTGCAG 3ˊ.

*SLC9A6_*Forward 5ˊ GAAGCTGTTAGGGGAAAT 3ˊ,

*SLC9A6_* Reverse 5ˊ CACTTATCTTTTGGGGTTGG 3ˊ,

### Molecular modeling and protein stability predictions

We utilized homology modeling to generate the protein structures without available crystal structures for the proteins of interest. The SWISS-MODEL server was employed for this purpose [35]. For SLC9A6, the CryoEM structure of the horse sodium/proton exchanger NHE9 in an inward-facing conformation (PDB ID: 6z3y) [36] was selected as the template, while the structure of the NaCT-Citrate complex (PDB ID: 7jsk) was used as the template for *SLC13A3* [37]. Both wild-type (wt) and mutated (mut) structures were generated for subsequent analysis. Model evaluation was performed using MolProbity [38], and a short molecular dynamics (MD) simulation was executed to optimize the structures. To assess the functional consequences and stability changes upon substitution (ΔΔG), we employed the DUET server [39, 40], which combines two complementary approaches (mCSM and SDM). Furthermore, we predicted the thermal stability changes (ΔΔG) arising from vibrational entropy changes (ΔΔS) using the Elastic Network Contact Model (ENCoM) server [41].

To investigate the effect of mutations on the overall structural dynamics of the proteins in comparison to their wild-type counterparts, we conducted MD simulations in two steps: a 100 ns MD simulation for refining and optimizing the models (wt and mut), followed by another 100 ns MD simulation to analyze the residual fluctuations with or without the reported mutations. All simulations were executed using AMBER 20 [42] following the same protocol as described elsewhere [20, 43].

## Results

### Clinical features of family A

Members of Family A resided in the Billitang area of Kohat, Khyber Pakhtunkhwa. The family was from Pashtun ethnic group that traditionally favours marriages between first or second cousins. One affected family member (**V:2**) was born to first cousins in the fifth generation of the presented family pedigree (Fig. 1a).

**Fig. 2:**
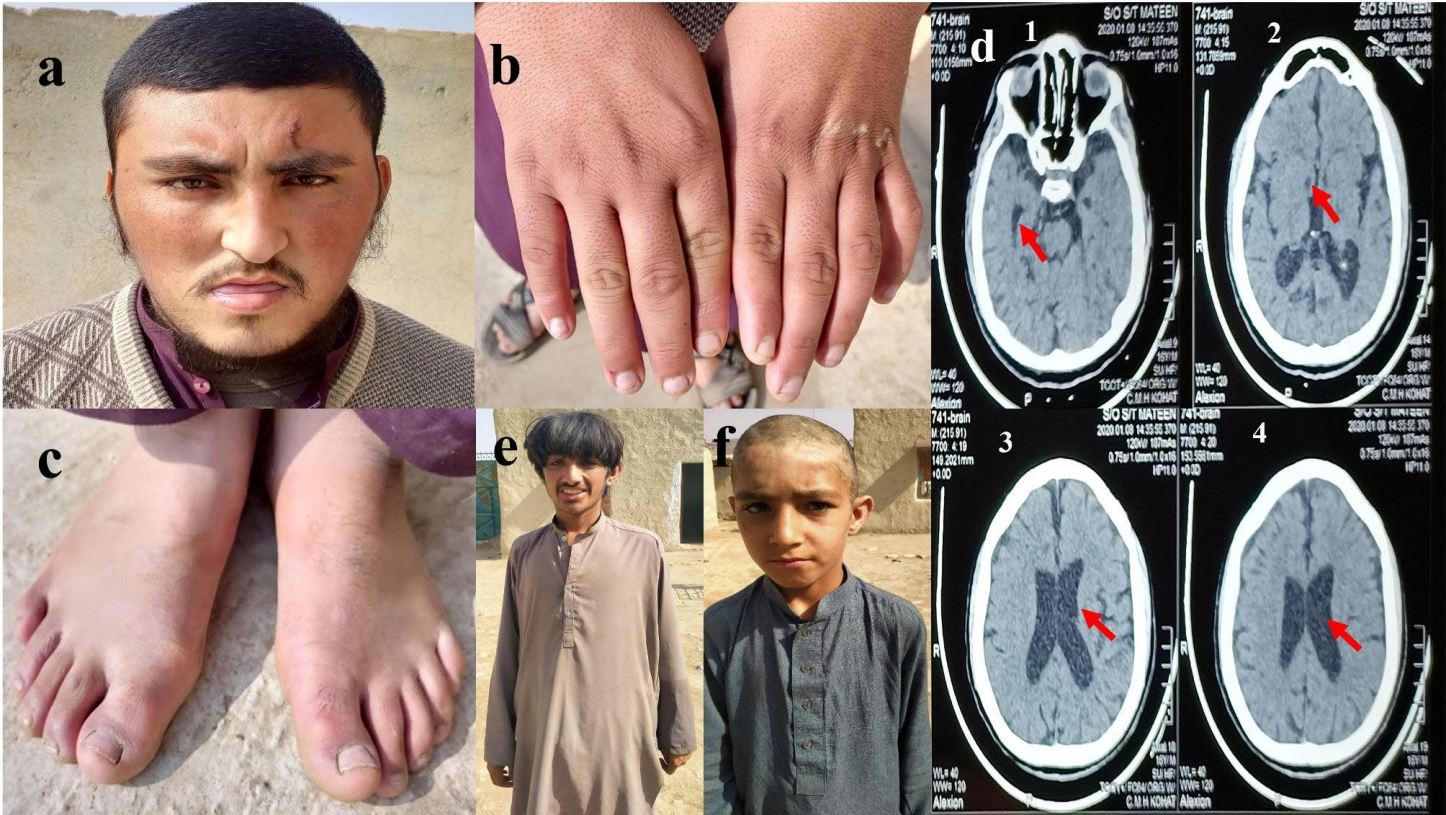
**(a, b, c)** Physical features presented by affected individual (V-2) of family A and **(d)** Computed tomography (CT) scan image of the affected individual (V-2) of family A indicative of multiple large CSF spaces within and around the brain suggestive of cerebral atrophy, **(e, f)** physical features presented by affected individuals (V-3 and V-4) of family B

The affected family member in Family A presented with clinical features associated with various physical and mental impairments, including short stature, unusual facial features, and enlarged thumbs and first toes. Besides vision problems, heart disease, kidney failure, tooth decay, and obesity are also indicators of the condition. Short and broad hands are typically associated with a large, sometimes spatulated thumb (Fig. 2a, b & c). In newborns, the palpebral fissures close in a highly distinctive way, creating a smiling appearance known as a “grimacing smile.“ On the other hand, no cases of spasticity, hypotonia, spasms, hypotonia, or deep tendon reflexes were observed. The CT scan revealed the presence of several large CSF spaces both inside and outside of the brain (Fig. 2d). The additional clinical description of patient in family A is summarized in table S1.

### Clinical features of family B

The members of Family-B lived in the North Waziristan region of Khyber Pakhtunkhwa. The family’s ethnicity was Pashtun, and they followed the cultural norm of marrying within the family. Two affected individuals (**V:3** and **V:4**) were born to first-cousin parents, making this the fifth generation in the family’s pedigree. Before our first visit for sampling, patient IV:7 appeared in the fourth generation and had already passed away for unknown reasons. No history of a genetic disorder had been detected in the family (Fig. 1b).

All the affected members of Family-B exhibited clinical features consistent with ID ranging from moderate to severe, including a large nose, disorganized speech, an open mouth, uncontrollable drooling, and abnormal eye movements. Affected kids usually have a bright disposition, with lots of smiles and giggles. The patients exhibited bizarre behaviour, developmental delay and deep tendon reflexes. However, no spasticity hypotonia and epileptic fits were observed. The patients’ dysmorphic facial features included an elongated, narrow face, a pointed chin, and a prominent nose, jawline, and ears (Fig. 2e & f). The additional clinical description of patients in Family B is summarized in table S2.

### Mutational analysis

**Fig. 3:**
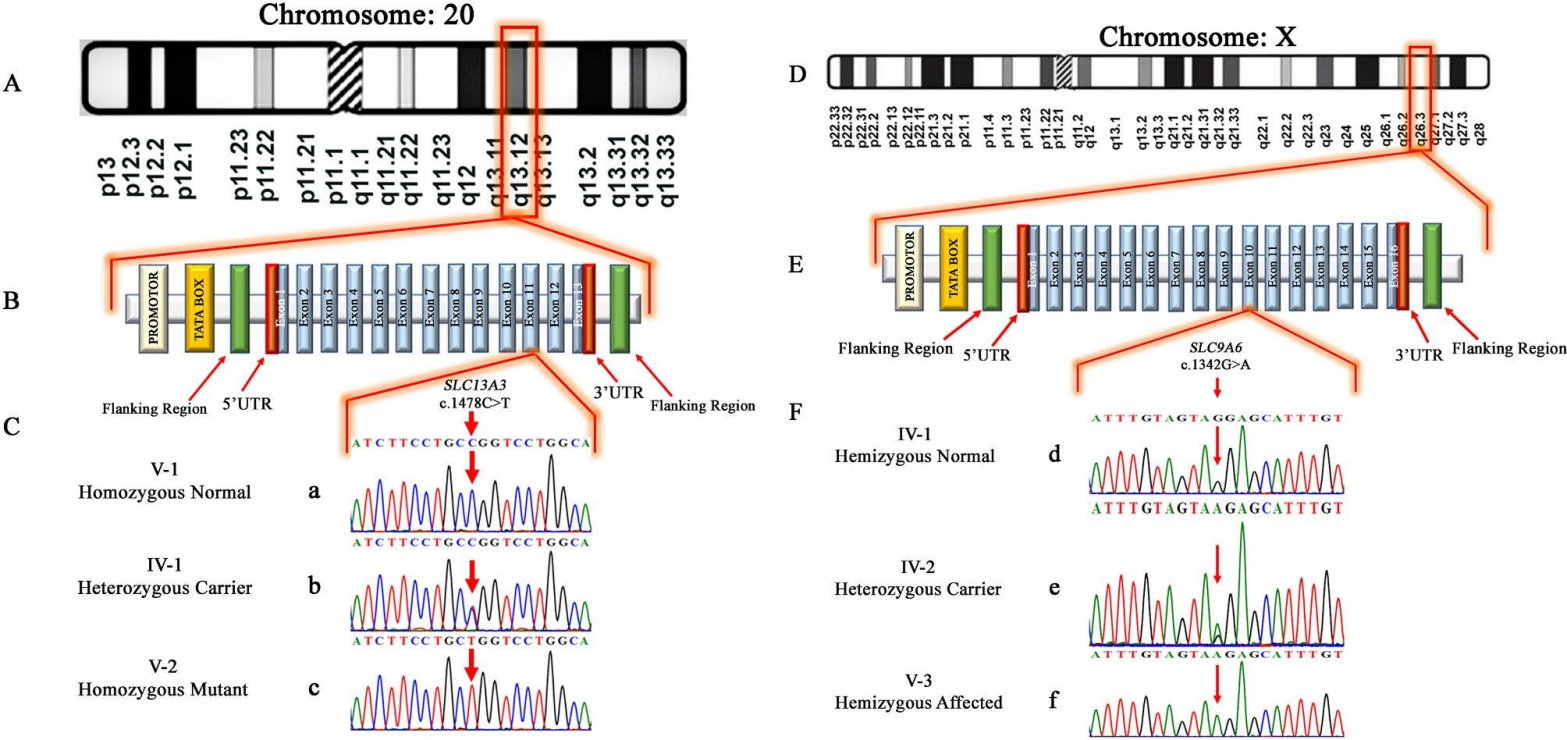
**(A)** Representing the location of *SLC13A3* (q13.12) at chromosome 20. **(B)** representing typical structure of the *SLC13A3* gene comprising 13 exons and also indicating the location of the missense variant (c.1478 C > T; p. Pro493Leu) in exon-11 **(C)** Chromatograms of homozygous unaffected son (V-1), heterozygous carrier father (IV-1) and homozygous affected son (V-2) of family A. **(D)** Location of *SLC9A6* (q26.3) at chromosome X. **(E)** the typical structure of *SLC9A6* gene comprising 16 exons, indicating the location of the missense variant (c.1342G > A; p. Gly448Arg) in exon-10. **(F)** Chromatograms of hemizygous unaffected father (IV-1), heterozygous carrier mother (IV-2) and hemizygous affected son (V-3) of family B

Genetic analysis using whole exome sequencing performed on DNA from an affected individual (V:2) from family-A revealed a novel homozygous missense variant (c.1478 C > T; p. Pro493Leu) in the exon-11 of *SLC13A3* gene (NM_022829.5). The segregation of this variant with disease phenotype was confirmed by Sanger sequencing. Zygosity analysis found both unaffected parents (IV:1 as well as IV:2) heterozygous carriers (c.1478 C/T), unaffected sibling (V:1) to be homozygous wild-type (c.1478 C/C), and the affected individuals (V:2) to be homozygous affected (c.1478T/T) for missense variant as mentioned earlier (Fig. 3a, b and c).

In family-B whole exome sequencing identified a novel missense variant (c.1342G > A; p. Gly448Arg) in the exon-10 of *SLC9A6* gene (NM_001042537.2). Sanger sequencing validated and confirmed the segregation of the variant with the disease phenotype in the family. Furthermore, the zygosity analysis determined that both the affected individuals (V:3 and V:4) were hemizygous affected (c.1342 A), mother and sister (IV:2 and V:1) were heterozygous carriers (c.1342G/A). At the same time, the father and phenotypically unaffected brother (IV:1 and V:2) were hemizygous wild-types (c.1342G) for the identified missense variant (Fig. 3d, e **and f**), consistent with an X-linked mode of inheritance.

### Structural elucidation

**Fig. 4:**
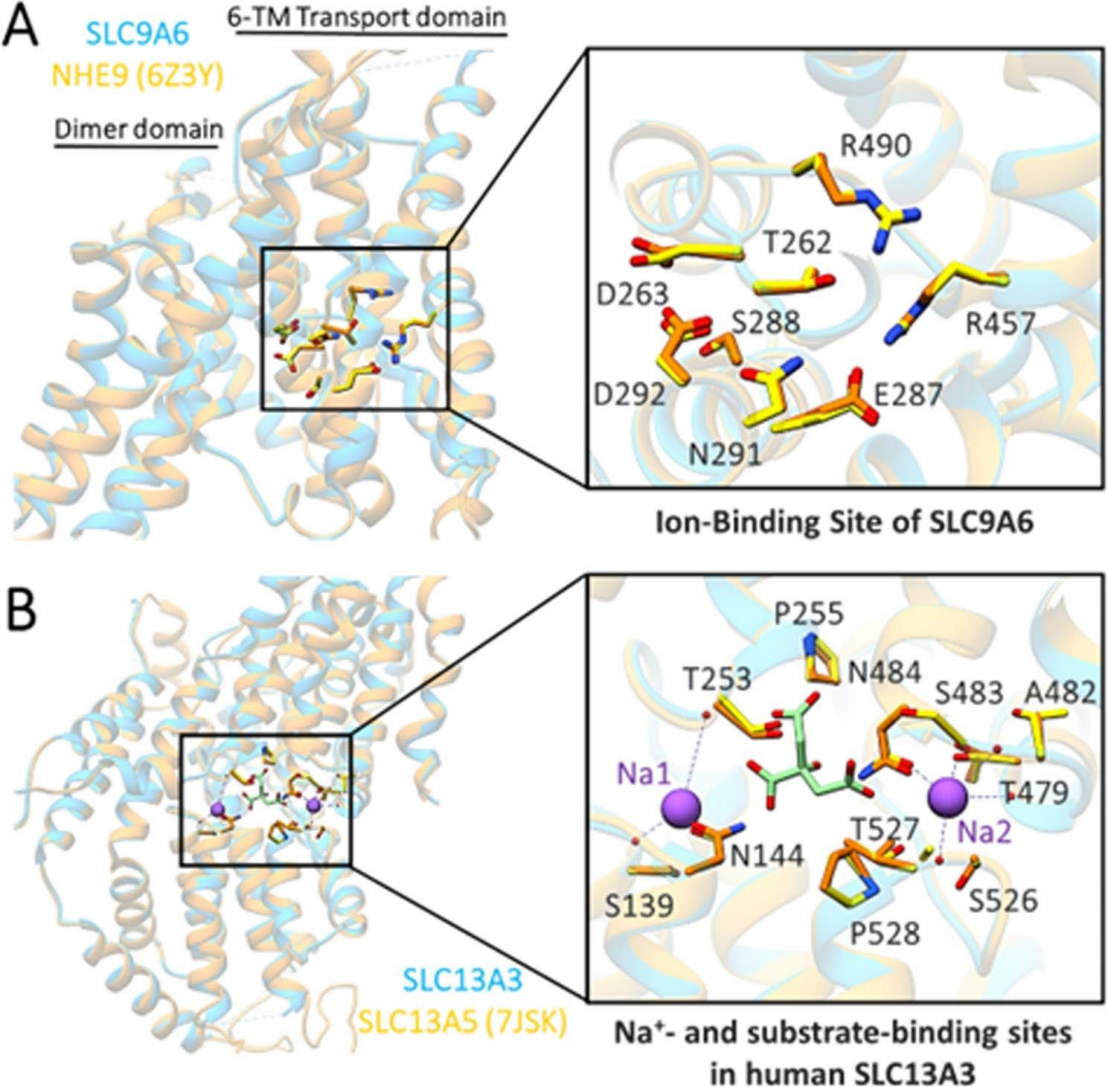
Comparative Binding Site Representations of SLC9A6 and SLC13A3. **(A)** Ribbon depiction of the SLC9A6 ion-binding site in the 6-transmembrane (TM) core transport domain, superimposed on NHE9 (PDB ID: 6Z3Y). A detailed view of the predicted ion-binding site of SLC9A6, with crucial residues displayed as yellow sticks and labeled accordingly. Corresponding residues in the NHE9 structure are indicated in orange. **(B)** Ribbon depiction of the SLC13A3 sodium and substrate binding site, superimposed on SLC13A5 (PDB ID: 7JSK). A detailed view of the predicted binding site of SLC13A3 is shown, with crucial residues represented as yellow sticks and labeled. Corresponding residues in the SLC13A5 structure are shown in orange

In our study, we identified the mutations Gly448Arg in *SLC9A6*, denoted as ‘SLC9A6_G448R mutant’, and Pro493Leu in *SLC13A3*, denoted as ‘SLC13A3_P493L mutant’. To investigate the structural implications of these mutations, homology models of *SLC9A6* and *SLC13A3* were generated using the SWISS-MODEL server. The *SLC9A6* model exhibited 68.47% sequence identity and 0.66 query coverage with the cryo-electron microscopy (cryo-EM) structure of NHE isoform 9 (*SLC9A9*) from Equus caballus (PDB ID: 6Z3Y). Both proteins share a conserved domain architecture, including a core ion-transport domain that is open toward the intracellular side. Near the base of the cavity lies the strictly conserved aspartate residue, Asp244 in SLC9A6 (Asp292 in *SLC9A9*), which is crucial for ion-binding and transport [44]. The ion-binding site and the negatively charged funnel are highly conserved across all NHE family members. Notably, the Gly448Arg mutation is located within the core ion-binding site, where key conserved residues such as Thr262, Asp263, Glu287, Ser288, Asn291, Asp292, Arg457, and Arg490 are present (Fig. 4).

On the other hand, the SLC13A3 model displayed 49.27% sequence identity and 0.91 query coverage with the cryo-EM structure of human NaCT in complex with citrate or a small-molecule inhibitor (PDB ID: 7JSK). The Pro493Leu mutation is positioned close to the substrate and Na^+^ binding sites. The Na1 is coordinated by conserved residues such as Ser139, Trp141, Gly252, and Asn144. Whereas, Na2 is surrounded by Thr479, Ala482 (Thr463 in SLC13A5), Ser526 (Ala507 in SLC13A5), and Asn484. The proximity of the Pro493Leu mutation to the sodium-binding site suggests a possible impact on the transporter’s function (Fig. 4).

Subsequently, the homology models were refined and optimized through 100 ns molecular dynamics (MD) simulations. The evaluation of the final models showed that 90.30% and 90.54% of the residues were in Ramachandran favoured regions for *SLC13A3* and *SLC9A6* models, respectively, indicative of reliable structural models.

### Protein stability predictions

The protein stability predictions for the mutated models of SLC9A6_G448R and SLC13A3_P493L, derived from the combined computational approach of mCSM and SDM, provided insights into the possible structural consequences of these mutations and their potential impact on the development of intellectual disability. For the SLC9A6_G448R mutation, the consensus prediction revealed a destabilizing effect on the protein structure, with a ΔΔG value of -0.138 kcal/mol. Conversely, in the case of the SLC13A3_P493L mutation, the consensus prediction from mCSM and SDM indicated a stabilizing effect on the protein structure, with a ΔΔG value of 0.446 kcal/mol. However, the Elastic Network Contact Model (ENCoM) server predicted a decrease in molecular flexibility for both mutations (ΔΔS_vib_ of -1.523 for SLC9A6_G448R and − 0.583 for SLC13A3_P493L), leading to contrasting effects on thermal stability (ΔΔG of 1.215 kcal/mol for SLC9A6_G448R, and 0.466 kcal/mol for SLC13A3_P493L). The SLC9A6_G448R mutation lies within the core ion-binding site, while the SLC13A3_P493L mutation is located close to the sodium binding pocket. These locations and the observed contrasting effects on protein stability and flexibility might potentially influence ion transport efficiency, thereby affecting the protein’s function and ultimately contributing to the development of intellectual disability. These preliminary findings were further investigated and validated through Molecular Dynamics (MD) simulations by analyzing the impact of these mutations on the overall structural dynamics and residual fluctuations.

### Molecular dynamics simulations interpretations

**Fig. 5:**
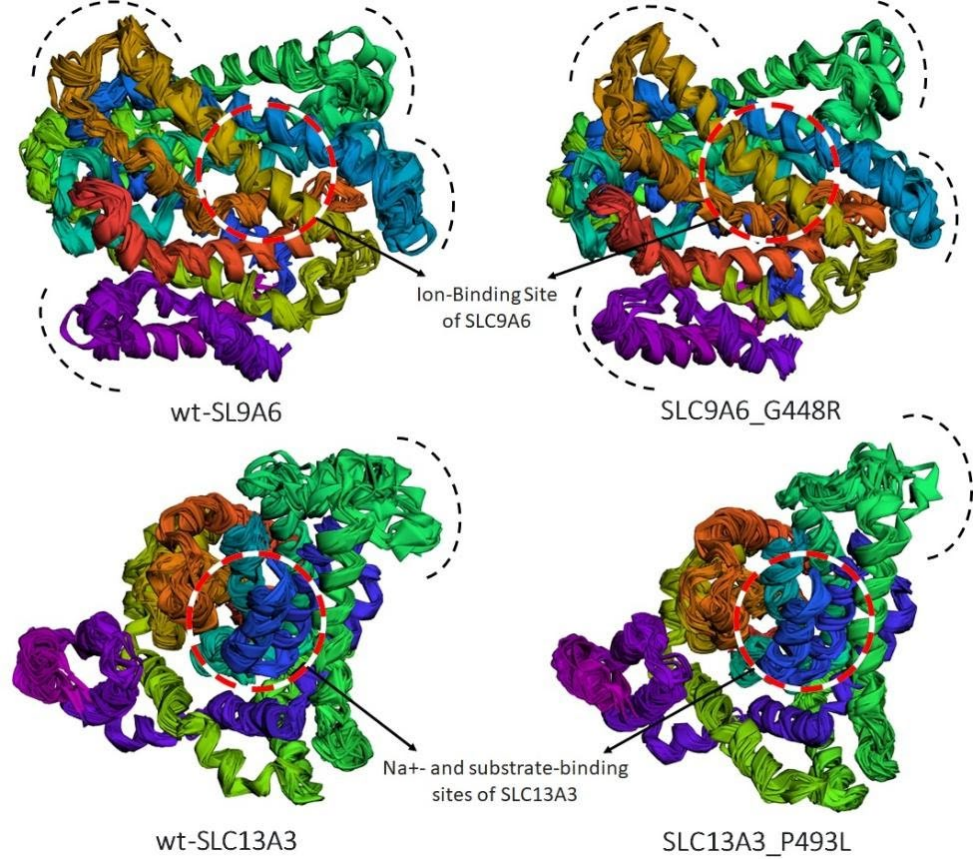
Structural Evolution of Wild-Type and Mutant Proteins Over 300 ns MD Simulations. The comparative progression of the wild-type and mutant protein models (SLC9A6_G448R and SLC13A3_P493L) during the 300 ns Molecular Dynamics (MD) simulation is illustrated. Snapshots taken every 30 ns offer a visual representation of changes in protein conformation and dynamics throughout the simulation period. Affected mutation sites are distinctly highlighted: for SLC9A6_G448R, the focus is on the intact ion-binding site, while for SLC13A3_P493L, it’s on the regions around the Na+- and substrate-binding sites, showcasing their dynamic fluctuations

A comprehensive 300 ns molecular dynamics (MD) simulations were conducted on the wild-type and mutant (SLC9A6_G448R and SLC13A3_P493L) protein models. Snapshots were taken every 30 ns throughout the simulation period to monitor the evolution of the protein structure and dynamics (Fig. 5). The initial phase of the simulation revealed a pattern of convergence for both the wild-type and mutant proteins, demonstrating consistent stability up to the 300 ns mark. This structural convergence provided a robust foundation for subsequent comparative analysis. Intriguingly, as the simulation progressed, the SLC9A6_G448R and SLC13A3_P493L mutant models displayed increased stabilization compared to their wild-type counterparts. These mutants maintained high stability throughout the simulation period, with minor fluctuations within an angstrom range. The enhanced structural stability could be indicative of significant functional changes. As the simulation advanced, the SLC9A6_G448R and SLC13A3_P493L mutant models showcased distinct stabilization patterns compared to their wild-type counterparts. Specifically, the ion-binding site in SLC9A6_G448R remained intact and stable over the simulation, while the regions around the Na+- and substrate-binding sites in SLC13A3 exhibited more pronounced fluctuations (Fig. 5).

Detailed structural insights revealed that substituting glycine for arginine at position 448 in SLC9A6_G448R led to more interactions due to a longer and more flexible side chain in arginine (Figure S1). The positively charged arginine guanidinium group can form hydrogen bonds and salt bridges with nearby residues, stabilizing the local environment around the mutation site. Similarly, substituting proline with leucine at position 493 in SLC13A3_P493L resulted in more interactions than proline in the wild-type protein (Figure S2). It can be attributed to leucine’s larger hydrophobic side chain, which promotes van der Waals interactions and hydrophobic packing with neighboring residues. These interactions remained consistent throughout the MD simulation time, explaining the increased stability in the mutant proteins compared to their wild-type counterparts. The mutations led to a more intact ion binding site, which may alter ion binding affinities or transport kinetics, impacting the overall function of the proteins. Consequently, this alteration in protein function due to the increased stability of the ion binding site establishes a potential link to the development of intellectual disability in the context of these mutations.

## Discussion

Solute carriers, or SLCs, are the most prominent family of transmembrane transporters responsible for the diffusion of nutrients, ions, metabolites, and drugs across cell membranes [45, 46] About 287 SLC genes are present in the human brain, and mutations or the resulting dysfunctions of 71 SLC genes have been linked to various neurodevelopmental disorders [47]. ARLIAK is an autosomal recessive condition that causes acute reversible neurologic degeneration during a febrile illness. Pathogenic SLC13A3 mutations cause acute reversible leukoencephalopathy (a heterogeneous set of disorders characterized by developmental defects or white matter degeneration) and ketoglutarate accumulation (ARLIAK) [9]. Dewulf et al. (2019) reported two patients with biallelic *SLC13A3* variants. After a bout of fever caused by a respiratory tract infection, one patient exhibited symptoms of acute neurological deterioration, including drowsiness, ataxia, and dysarthria. The other patient had febrile tonsillitis and relapsed six years later due to a febrile respiratory tract infection, presenting with drowsiness, poor contact, dysarthria, peripheral motor abnormalities, and global hypotonia. After a short supportive treatment, they returned to nearly complete health. It is the first study to link a-ketoglutarate accumulation and reversible leukoencephalopathy to biallelic variants of *SLC13A3* [9]. Proximal tubule cells express NaDC3 mostly at their basolateral membrane, which is thought to be important in importing dicarboxylates from the interstitial space [48]. Previous functional studies of *SLC13A3* variants significantly reduced the capacity of NaDC3 to transport the three substrates due to loss of function that may lead to physiological conditions [9].

Christianson syndrome (CS) is a neurodevelopmental and degenerative X-linked intellectual disability disorder with a growing number of confirmed cases. The enzyme NHE6 controls the pH balance and trafficking of recycled endosomes [49]. Since this ion transporter is highly expressed in the brain, it may help to explain the wide variety of neural phenotypes seen in CS. Therefore, the discovery of specific NHE6-dependent receptors contributes to our understanding of the mechanism of neuronal dysfunction in CS. Transport of vesicles containing AMPA receptors to and from the postsynaptic membrane may be disrupted if endosomal acidification is impaired or absent due to a SLC9A6 gene mutation [50]. A large number of CNS neurons rely on the brain-derived neurotrophic factor (BDNF)/tropomyosin receptor kinase B (TrkB) neurotrophic signaling pathway for proper dendrite development [51]. This over-acidification, in conjunction with the accelerated degradation of TrkB, can disrupt endosomal BDNF/TrkB signaling, leading to the death of neuronal axons and dendritic branches [52]. Microtubule-associated proteins (MAP) are crucial to average brain growth and development. Mature neurons rely profoundly on tau protein primarily associated with microtubules. Different tau isoforms are expressed at specific times and places during brain development [53], indicating that tau isoform regulation is crucial for proper brain development [54]. The NHE6 protein, encoded by *SLC9A6*, is primarily found in early and recycling endosomes, where it participates in endosomal trafficking, signaling, and the regulation of luminal pH [18, 55–57]. In a previous study, western blot analysis of two variants (p.T521Yfs*23, p.H203Lfs*10) revealed significantly decreased mRNA levels and normal NHE6 protein [58, 59]. Western blot analysis of these variants further revealed that both variants caused the total loss of function of NHE6 protein and confirmed that CS is mainly caused by NHE6 loss of function [59].

Our current genetic study enrolled two consanguineous Pakistani families with Pashtun ethnicity segregating intellectual disability. Genetic analysis in family-A revealed a novel homozygous missense variant (c.1478 C > T; p.Pro493Leu) in the exon-11 of *SLC13A3* gene (NM_022829.5), while in family-B, a novel missense variant (c.1342G > A; p.Gly448Arg) in the exon-10 of the *SLC9A6* gene (NM_001042537.2) was identified.

NaDC3 is a plasma membrane cotransporter encoded by the *SLC13A3* gene and found in the kidney, brain, liver, placenta, and eye [60, 61]. It is essential for cell nutrition and detoxification because it transports citric acid cycle intermediates (succinate and a-ketoglutarate) and other critical metabolic compounds (glutathione, mercapto succinate, and NAA) into the cell [6, 7]. Abnormalities found in the central nervous system (CNS) may have a genetic basis, as pathogenic variants in *SLC13A3* are known to reduce the transport capacity for a-ketoglutarate, succinate, and NAA, suggesting a loss-of-function mechanism [9]. NaDC3 is localized in the kidney’s luminal membrane by absorbing dicarboxylates from the glomerular filtrate. Therefore, urinary a-ketoglutarate accumulation may result from impaired NaDC3 function. As a result of the c.1478 C > T missense variant, a corresponding substitution was found in our patient (p.pro493leu). These variants may cause disease by altering the structure and function of NaDC3 [48].

Clinical manifestations attributable to *SLC9A6* mutations mirror developmental and progressive pathophysiology [11]. *SLC9A6* knockout mouse models revealed abnormalities in endosomal-lysosomal function and cholesterol accumulation in specific neuronal populations, similar to those in primary lysosomal storage diseases [62]. In addition, the epileptic phenotype and decreased seizure threshold were observed in *SLC9A6*/ mutant female mice and *SLC9A6*^−^/^0^ mutant male mice [18]. Failure of axonal and dendritic branching, resulting in impaired neuronal connectivity, may contribute to cognitive and language impairment in children with *SLC9A6* mutations [63]. Putative protein-truncating early frameshift, nonsense, or splicing mutations, as well as some missense or intra-frame deletions that may be residual protein, appear to be the most common types of *SLC9A6* mutations in CS patients [11].

Through state-of-the-art in silico studies, we explored the impact of these mutations, G448R in SLC9A6 and P493L in SLC13A3, on protein stability and function and their potential link to the development of intellectual disability. Protein flexibility plays a crucial role in determining the functional properties of a protein, as it is often associated with conformational changes and dynamic interactions required for proper function, ligand binding, and protein-protein interactions [64, 65]. Alterations in protein flexibility can lead to changes in protein function, either by affecting the binding affinity for substrates, modulating the protein’s activity, or impacting its interactions with other proteins or molecules [66]. In our study, the consensus stability predictions for the SLC9A6_G448R and SLC13A3_P493L mutations revealed contrasting effects on protein stability, with SLC9A6_G448R showing a destabilizing effect and SLC13A3_P493L displaying a stabilizing effect. However, both mutations were predicted to decrease molecular flexibility, potentially leading to functional consequences. It has been reported that proteins’ stability and flexibility changes can affect protein function and be involved in disease development [67]. Molecular dynamics simulations provided further insights into the structural effects of these mutations. The mutants exhibited increased stability compared to their wild-type counterparts, particularly in the regions around the binding pockets. This increased stability can potentially impact ion binding and transport, as a more rigid binding site might alter the protein’s ability to undergo the conformational changes required for efficient ion transport [64, 68]. It’s essential to address a pivotal observation regarding the seeming discrepancy between the protein stability predictions from mCSM, SDM, and the insights gained from MD simulations. While mCSM and SDM offered a destabilizing perspective for the SLC9A6_G448R mutation, MD simulations portrayed increased stability. This difference can be attributed to the inherent methodologies of the predictive tools versus the dynamic temporal representations given by MD simulations. Based on vast datasets, mCSM and SDM’s generalized predictions might not capture the unique, localized interactions evident in a more fluid MD environment. The arginine substitution in SLC9A6_G448R led to specific stabilizing exchanges in MD simulations, highlighting the importance of using diverse methods for comprehensive understanding. This observed stability might impact protein function even if the overall stability is decreased, underscoring that stability only sometimes translates directly to functional efficacy.

These observed stability and molecular flexibility changes could be linked to either gain or loss of function in the proteins. In intellectual disability, both gain and loss of function can potentially contribute to the disease’s development by disrupting normal cellular processes or signaling pathways [69]. Therefore, the mutations investigated in this study could impact protein function by altering the ion binding or transport dynamics, ultimately leading to intellectual disability. Future experimental studies should be conducted to validate these in silico findings and further investigate the molecular mechanisms underlying the link between these mutations and intellectual disability.

### Electronic supplementary material

Below is the link to the electronic supplementary material.

Supplementary Material 1

Supplementary Material 2

Supplementary Material 3

## Data Availability

The raw data (sequence, photographs, and pedigrees) is stored in the password-protected computer at Kohat University of Science and Technology, Kohat, and is available upon request. We have submitted the variant data to ClinVar (https://www.ncbi.nlm.nih.gov/clinvar/), and the accession numbers SCV003925774 and SCV003925775 have been assigned.
