# Supplementary material for: Structural and functional implications of SLC13A3 and SLC9A6 mutations: an in silico approach to understanding intellectual disability
Source: BMC Neurol. 2023 Oct 4;23:353. doi: 10.1186/s12883-023-03397-y (PMC10548666; doi:10.1186/s12883-023-03397-y)
Supplement: Supplementary file 1 — Supplementary Material 1 [file 12883_2023_3397_MOESM1_ESM.doc]

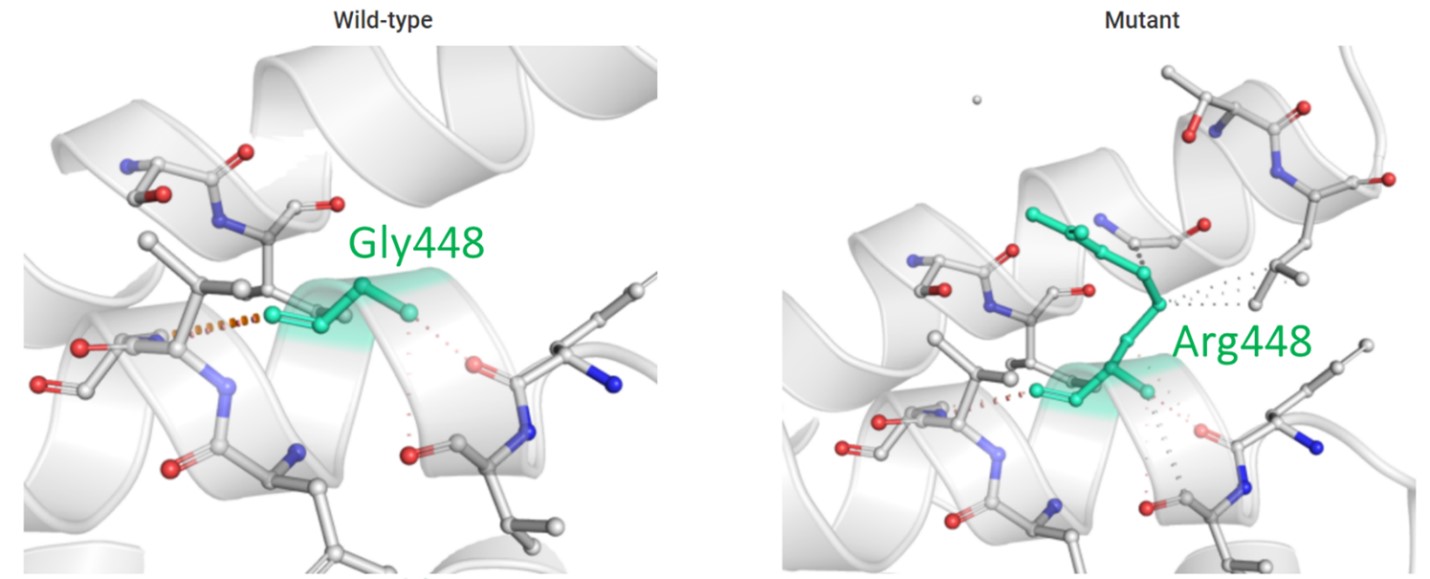


**Figure S1:** Comparative Depiction of Wild-Type and Mutant Residues in SLC9A6. The representation of the wild-type and mutated residue (Gly448Arg) within the SLC9A6 model. Both the wild-type and mutant residues are colour-coded in light green and depicted as stick models. The surrounding residues, crucial for interaction, are also represented as stick models to showcase their spatial relationship with the mutated residue.

**
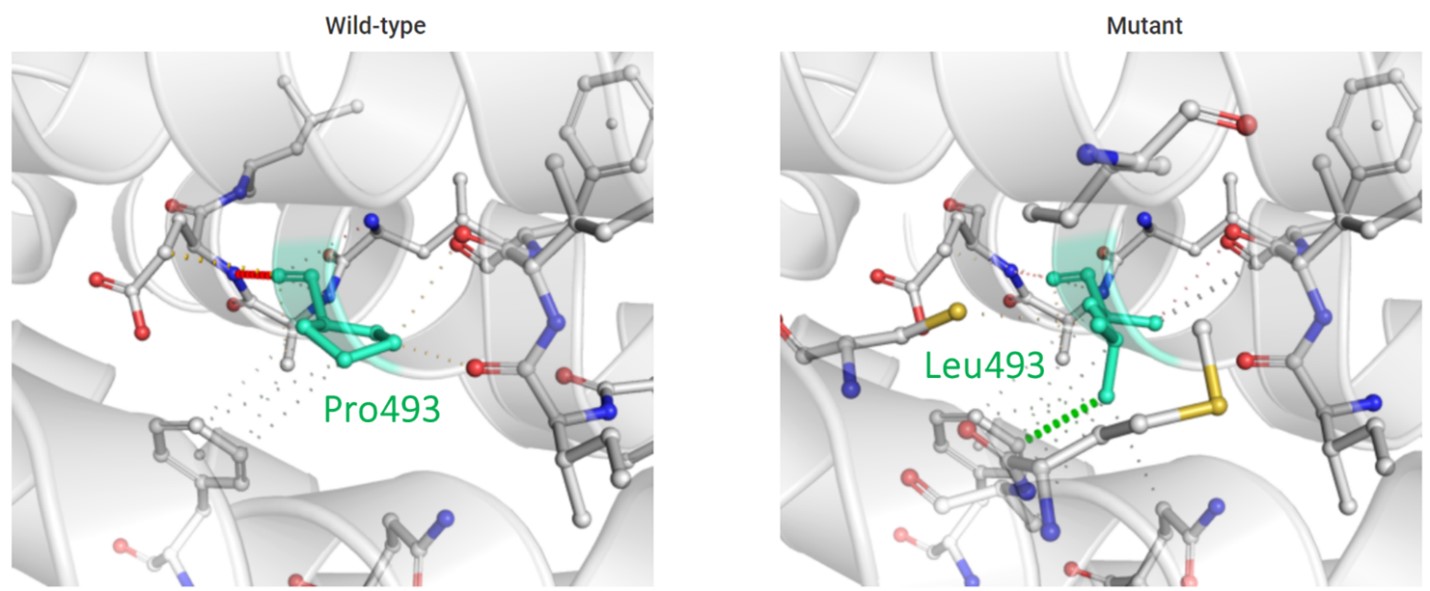
**

**Figure S2:** Comparative Depiction of Wild-Type and Mutant Residues in SLC13A3. The representation of the wild-type and mutated residue (Pro493Leu) within the SLC13A3 model. Both the wild-type and mutant residues are colour-coded in light green and depicted as stick models. The surrounding residues, crucial for interaction, are also represented as stick models to showcase their spatial relationship with the mutated residue.
