# Supplementary material for: Structural and functional implications of SLC13A3 and SLC9A6 mutations: an in silico approach to understanding intellectual disability
Source: BMC Neurol. 2023 Oct 4;23:353. doi: 10.1186/s12883-023-03397-y (PMC10548666; doi:10.1186/s12883-023-03397-y)
Supplement: Supplementary file 2 — Supplementary Material 2 [file 12883_2023_3397_MOESM2_ESM.docx]

**Table S1:** Summary of molecular genetics and key clinical findings in **Family A**.

| **Family A V:2** | | |
| --- | --- | --- |
| **Molecular Genetics Summary** | | |
| Genomic position | g. 109831 | |
| *SLC13A3* c. position | c.1478C>T | |
| *SLC13A3* p. position | p.(Pro493Leu) | |
| CADD_Phred | 29.7 | |
| PolyPhen-2 | Probably Damaging | |
| SIFT | Not Tolerated | |
| MutationTaster | Disease Causing | |
| gnomAD v.3.1.2 Frequency | 0.0000131 | |
| gnomAD v.2.1.1 Frequency | 0.0000248 | |
| PhyloP100 | 9.799 | |
| **Clinical Summary** | | **Phenotypic Features Reported Previously (%)** |
| Developmental delay | + | 100 |
| Microcephaly | - | 0 |
| Seizures | + | 100 |
| Intellectual disability | + | 100 |
| Spasticity | - | - |
| Hypotonia | - | - |
| Deep tendon reflexes | - | - |
| Behavioural abnormalities | + | 100 |
| Prominent Nose | + | 100 |
| Long Face | - | 20 |
| High Nasal Bridge | + | 100 |
| Ptosis | + | 100 |
| Micrognathia | - | - |
| Language Skills | Poor | 80 |
| Abnormal brain CT Scan | + | 100 |
| Hearing loss | - | 40 |

Transcript NM_022829.5, “-“ means normal, “+” means abnormal, “?” means uncertain
