# Supplementary material for: Structural and functional implications of SLC13A3 and SLC9A6 mutations: an in silico approach to understanding intellectual disability
Source: BMC Neurol. 2023 Oct 4;23:353. doi: 10.1186/s12883-023-03397-y (PMC10548666; doi:10.1186/s12883-023-03397-y)
Supplement: Supplementary file 3 — Supplementary Material 3 [file 12883_2023_3397_MOESM3_ESM.docx]

**Table S2:** Summary of molecular genetics and key clinical findings in **Family B.**

| **Family B V:3/ V:4** | | |
| --- | --- | --- |
| **Molecular Genetics Summary** | | |
| Genomic position | 31312G>A | |
| *SLC9A6* c. position | c.1342G>A | |
| *SLC9A6* p. position | p.Gly448Arg | |
| CADD_Phred | 26.1 | |
| PolyPhen-2 | Benign | |
| SIFT | Not Tolerated | |
| MutationTaster | Disease Causing | |
| gnomAD v.3.1.2 Frequency | 0 | |
| gnomAD v.2.1.1 Frequency | 0 | |
| PhyloP100 | 7.621 | |
| **Clinical Summary** | | **Phenotypic Features Reported Previously (%)** |
| Developmental delay | +/+ | 100 |
| Microcephaly | -/- | 20 |
| Seizures | -/- | 70 |
| Intellectual disability | +/+ | 100 |
| Spasticity | -/- | - |
| Hypotonia | -/- | 60 |
| Deep tendon reflexes | +/+ | 80 |
| Behavioural abnormalities | +/+ | 100 |
| Prominent Nose | +/+ | 80 |
| Long Face | +/+ | 100 |
| High Nasal Bridge | -/- | - |
| Ptosis | +/+ | 70 |
| Micrognathia | -/- | - |
| Language Skills | poor | 85 |
| Abnormal brain CT Scan | +/+ | 100 |
| Hearing loss | -/- | - |

Transcript 001042537.2, “-“ means normal, “+” means abnormal, “?” means uncertain
